# Women's experiences of their osteoporosis diagnosis at the time of diagnosis and 6 months later: A phenomenological hermeneutic study

**DOI:** 10.3402/qhw.v9.22438

**Published:** 2014-02-21

**Authors:** Carrinna Hansen, Hanne Konradsen, Bo Abrahamsen, Birthe D. Pedersen

**Affiliations:** 1 Research Unit of Nursing, Faculty of Health Sciences, Institute of Clinical Research, University of Southern Denmark, Odense M, Denmark; 2 Gentofte University Hospital, Hellerup, Denmark; 3 Faculty of Health Sciences, Institute of Clinical Research, University of Southern Denmark, Odense M, Denmark

**Keywords:** Interpretation, interview, nursing research, Ricoeur, qualitative research

## Abstract

This paper describes a phenomenological hermeneutic study of experiences of women who were recently diagnosed with osteoporosis. The research objective was to investigate women's experiences of living with osteoporosis during the first 6 months after diagnosis when treatment was first prescribed. Fifteen women were included in the study. The inclusion criteria were a DXA scan at one of the two hospitals showing a T-score below −2.5 (lower back or hip), age 65 years or older; no previous known osteoporotic fracture; at least one of the known risk factors for osteoporosis; and prescription of anti-osteoporotic treatment. Exclusion criteria were previous diagnosis of osteoporosis or previous treatment with anti-osteoporotic medication. Data were collected through in-depth interviews shortly after diagnosis and 6 months later. The performed analyses were inspired by Paul Ricoeur's theory of interpretation of texts comprising three levels: naïve reading, structural analysis, and critical interpretation and discussion. Three key themes emerged: 1) *being diagnosed*, 2) *being prescribed medical treatment*, and 3) *being on the path of learning to live with osteoporosis*. The findings suggest a need for improved support for the patients to gain understanding of their diagnosis and the risk of osteoporotic fracture as well as to learn to live with osteoporosis. The study highlights new health promotion areas for targeting interventions at newly diagnosed patients, helping them accept and interpret the diagnosis, and the medical treatment.

Osteoporosis has become a major worldwide challenge because of an aging population with increasing number of chronic diseases (Kanis et al., 2013; World Health Organization, 2003). One in three European women over age 50 will experience osteoporotic fractures, as will one in five men. In Denmark, osteoporosis is estimated to afflict >40% women and 18% men aged 50 years and over (Vestergaard, Rejnmark, & Mosekilde, 2005). In the current study, we have chosen to focus on women because they are more often affected by osteoporosis.

Early detection and treatment are essential in prevention of deterioration and disability, improvement of prognosis and quality of life, and prevention of premature death (Abrahamsen, Van, Ariely, Olson, & Cooper, 2009; Cockerill et al., 2004; Kanis et al., 2013; Weston, Norris, & Clark, 2011).

Literature regarding lived experience of osteoporosis published before February 2013 was systematically searched using CINAHL, ERIC, EMBASE, and PubMed databases. Osteoporosis was used as the MESH-term combined with the following terms: lived experience(s), life experiences, and phenomenological and qualitative studies/research. Inclusion criteria were qualitative studies investigating patients’ experiences or thoughts related to osteoporosis. The search was limited to languages such as English, German, Norwegian, Swedish, or Danish.

Research has shown that many patients find it difficult to perceive and interpret the diagnosis, their current risk, and managing everyday life with osteoporosis. One reason may be that some patients are diagnosed before they experience osteoporotic fractures. However, most individuals with osteoporosis are unaware of the disease until bone fracture occurs, as osteoporosis is usually an asymptomatic condition (Nielsen, Huniche, Brixen, Sahota, & Masud, 2013; Weston et al., 2011). Several qualitative studies have demonstrated that a diagnosis of osteoporosis may lead to psychological and physical consequences for the individuals, and it may affect quality of life (Nielsen et al., 2013; Reventlow, 2007; Reventlow & Bang, 2006; Reventlow, Hvas, & Malterud, 2006; Weston et al., 2011; Wilkins, 2001a). Studies have shown that an awareness of osteoporosis and fracture risk may cause worries and uncertainty. Elderly women's perceptions of osteoporosis and fracture risk as possible threat to health has been found to be influenced by stereotypes of bodily decay due to aging (Hvas, Reventlow, Jensen, & Malterud, 2005; Reventlow & Bang, 2006; Reventlow et al., 2006). There are diverse ways in which women with osteoporosis perceive themselves and manage their aging and chronic illness (Roberto & Reynolds, 2001; Wilkins, 2001b). A systematic review of 22 papers evaluating health behavior and health belief of osteoporosis (McLeod & Johnson, 2011) has found that structural and psychological determinants of health behavior need to be understood in order to better understand and manage the disease. In the past decade, there has been a growing focus on the effectiveness of person-centered interventions facilitating health behavior change. Showing that individuals generate strategies or relapse to earlier life style in relation to health behavior changes very early in the trajectory, findings indicate that it is a combination of events which may lead to changes (Ryan, 2009; Tollen, Kamwendo, & Ivarsson, 2011). Although it has been found that educational programs may increase patients’ knowledge of osteoporosis and enhance adherence to treatment (Ryan, 2009; Sedlak, Doheny, Estok, Zeller, & Winchell, 2007), it is also argued that educational programs and increased knowledge in itself do not lead to change in health behavior (Ryan, 2009). According to fracture preventive behavior, it has been found to be a motivational process through which healthy risk awareness can be developed. The knowledge of fracture risk in everyday life may be handled well by some patients, but may affect others in a degree which could limit their daily activities (Hjalmarsson, Strandmark, & Klässbo, 2007; Nielsen et al., 2013).

In light of these studies, it seems to be important to further illuminate the individual's experience of osteoporosis in the early stages of the diagnosis and to search for a deeper understanding of the dimensions that influence the individual's life with osteoporosis. To our knowledge, very few studies have explored stories of experiences of osteoporosis in early stages after diagnosis. Therefore, the aim of the current study was to investigate women's experiences of living with a new osteoporosis diagnosis during the first 6 months after diagnosis when fracture preventive treatment had been prescribed.

## Method

The study used a qualitative method and data were obtained by open interviews (Kvale & Brinkmann, 2009; Malterud, 2001) and was scientifically directed within a phenomenological hermeneutic approach guided by Paul Ricoeur's theory of interpretation of text (Lindseth & Norberg, 2004; Pedersen, 1999; Ricoeur, 1976). This approach allowed us to gain insight into women's experiences. Distanciation was obtained because the interviews were verbatim and fully transcribed and thereby appeared as a text. According to Ricoeur, interpreting a text means to see something new in what is already taken for granted (Ricoeur, 1973).

All women 65 years or older who attended DXA scan at one of the two participating hospitals were asked to consider participation in the study. They were included consecutively according to inclusion criteria from January to April 2011. The inclusion criteria were a DXA scan showing a T-score below −2.5 (lower back or hip) which indicates osteoporosis (Kanis et al., 2013), no previous known osteoporotic fracture, at least one of the known risk factors for osteoporosis (Kanis et al., 2013), and prescription of anti-osteoporotic treatment. Exclusion criteria were previous diagnosis of osteoporosis or previous treatment with anti-osteoporotic medication. The women were contacted by a health care professional, given an information letter, and asked to consider participation in the study. Those who agreed to participate gave their names and phone numbers and were contacted shortly after by the researcher. All patients who met the inclusion criteria were included; one informant wished no further participation when contacted prior to the second interview because of personal reasons.

Fifteen women (mean age 71.9 years, range 65–79) with osteoporosis were included. Throughout the study, analyses and interpretations were discussed with fellow researchers during all three levels of interpretation to enhance trustworthiness. Involvement of multiple researchers is recommended when conducting qualitative research, because this might strengthen the design of the study. During the analyzing process and interpretation, multiple researchers may supplement and contest each other's statements which may enrich and qualify the analysis (Kvale & Brinkmann, 2009; Malterud, 2001).

Data were obtained through individual interviews (Kvale & Brinkmann, 2009; Malterud, 2001), undertaken by the first author in the spring and fall 2011. The first interview took place shortly after the diagnosis; the second interview was about 6 months later. These points of time are chosen because studies have shown that patients’ medical adherence stabilizes around 6 months after initiation (Kanis et al., 2013) and adjustments to live with a chronic condition are often lengthy (Holloway, 2007). The interviews were determined by the individual informant which led to most interviews taking place in the informants’ private homes. Three informants chose to give interviews at the hospital. They were telling about their experiences and reflections upon life with osteoporosis. The interviews lasted for 21–72 min (mean 45.38 min); they were tape-recorded and transcribed verbatim and fully. An open interview guide was used during the interviews to ensure consistency and encourage openness and flexibility during the interviews and the interview guide was adjusted between interview-rounds (Kvale & Brinkmann, 2009). The opening question was: “Please tell about your experiences of living with osteoporosis.” The informants were thus encouraged to tell stories of their personal experiences of everyday life when living with osteoporosis. The approach to data collection was open and narrative with the interviewer asking supplementary questions to ensure rigor in capturing the informants’ perspective (Kvale & Brinkmann, 2009). The second interview was initiated by the interviewer saying: “Now I am very excited to hear how you have been doing since last time I saw you.”

The study was approved by the Danish Data Protection Agency (J.no. 2012-41-0875) and the National Committee on Health Research Ethics (J.no. H-C-FSP-2011_01). The informants were given written and oral information and verbal informed consent was obtained before each interview (World Medical Association, 2000).

## Data analysis

The text was analyzed as a whole – not referring to interview round one or two or specific individuals, which is in line with Ricoeur's theory of interpretation of texts (Ricoeur, 1973). The interpretation of data in this study had three levels: a naïve reading, a structural analysis, and a critical analysis and discussion.

*Naïve reading* is the first reading and re-reading of the text in order to grasp its meaning as a whole. The interpreter tries to read the text with a phenomenological attitude, with as much openness as possible, thus allowing the text to speak. The naïve reading is regarded as the first presumption (Lindseth & Norberg, 2004; Pedersen, 1999).

*A structural analysis* was carried out to validate and adjust the naïve interpretation and to reach a deeper understanding of the experiences of living with osteoporosis. According to Ricoeur, to understand a text is to follow its movement from what it says to what it talks about (Lindseth & Norberg, 2004; Pedersen, 1999). Thus, the structural analysis is describing units of meaning (what is said) and next identifying and formulating units of significance (what is talked about) leading to emission of key themes, subthemes, and patterns (Pedersen, 1999).

*Critical interpretation and discussion* is the final level of analysis; explanation and comprehension as a discussion of the findings by relating to the quotes from the interviews and to the text as a whole, incorporating relevant literature and other research findings (Pedersen, 1999).

## Findings

Through the naïve reading, insight into what the text is about and experiences related to the diagnosis process were described in the following terms: to be taken seriously, to feel taken care of, and to accept and become aware of osteoporosis. Furthermore, the experience of having a diagnosis which required medical treatment was described as a significant event, including both the trust in the diagnosis and medical treatment and uncertainty, worries, and anxiety. Additionally, informants’ descriptions of their life with osteoporosis showed that they perceived a necessity to adapt to living with osteoporosis by adapting their views of physical activity and diet changes as well as newly adjusted lifestyle. The naïve reading showed experiences of living with osteoporosis to be closely related to the patient trajectory of the diagnosis, the interpreted meaning of the medical treatment, and reflections regarding living with osteoporosis.

Through the structural analysis three key themes emerged: 1) *being diagnosed*, 2) *being prescribed medical treatment*, and 3) *being on the path of learning to live with osteoporosis*. An example of the structural analysis is exemplified in Table I. The themes will subsequently be described in interpretation; the statements are quotes and a textual illustration of the opening toward the interpretation (Ricoeur, 1976).

**Table I:** An example of the structural analysis and themes—To illustrate the opening of interpretation.

**Meaningful units** what is said “Quotes”	↔	**Significant units** what is spoken about (primary interpretation)	↔	Subthemes	↔	**Themes** emissions of key themes

he [the chiropractor] had made a tape for my physician, but of course she [the physician] would not consider looking at it...		Not being taken seriously by the GP but have to be an advocate for one's own health	to be taken seriously			Being diagnosed
I'd rather be affiliated with a hospital. I'm probably a little authoritarian. Normally I don't have that much confidence in the system, but that is nevertheless where I feel safest		Trust toward the health care system as a matter of being authoritarian	acceptance			
I am absolutely hysterical with medication, pills and such. Because, the pills helps treating one thing but it also harms something else. I cannot fill myself with all that crap		Having a strong and general attitude toward medication	decision against the medical treatment			Being prescribed medical treatment
I have many thoughts, oh my God, is it harmful... do I make the right choice? I find it very difficult to choose		Having worrisome thoughts and feelings of responsibility of making the right choice	decision to pursue the medical treatment			
I have always looked forward and I still do; it cannot do any good to put yourself away and sink into a chair because you have something		Attitude to life: to maintain hope and courage	the need to adapt			Being on the path of learning to live with osteoporosis
Even the days when it feels like I don't want to go for a walk – I'll do it anyway. I love to sit in the shade, but I am starting to sit more in the sun		Changing habits due to the need to adapt to life with osteoporosis	lifestyle changes			

### Being diagnosed

Experience of *being diagnosed* was interpreted as a process because several informants described it as extending over a longer period of time, sometimes years, during which the diagnosis was uncertain or unknown. The informants were generally associated being diagnosed to previous experiences of contacts with the health care system. The key theme was elaborated by two subthemes: *to be taken seriously* and *acceptance of the diagnosis*.

*To be taken seriously* was described as a central aspect during the diagnosis as well as afterwards. It affected and contributed to the relationship with the physician by promoting a feeling of care and trust. The experience of being taken seriously was described as being fostered by the physician's awareness of the increased risk of osteoporosis (due to previous illness or hereditary predisposition) and the physician's actions according to suspected osteoporosis. The subtheme was found in the text in both a positive and a negative context.

In general, it was found that the physician's action upon the increased risk, uncertainty, or new symptoms, created a feeling of being taken seriously:

76-year-old former high school teacher “I feel that health care providers take it seriously”

This informant described how well she felt taken care of by the health care professionals: the chief physician at the hospital who referred her to a DXA scan; the medical laboratory technologist who was highly skilled, caring, and informative; and the general practitioner (GP) who afterward explained once again about the medical treatment.

Descriptions of the absence of the feeling of being taken seriously were also commonly found in the text. These descriptions were in terms of needing to be a persistent advocate for one's own health and having to convince the physician of the need for a thorough examination. Among others, this appeared in the text as a description of the physician's apparent rejection to accept information from other therapists:

74-year-old former preschool teacher “he [the chiropractor] had made a tape for my physician, but of course she [the physician] would not consider looking at it...”

The irony by which this sentence ends, elucidates the feeling that a struggle to be taken seriously was experienced, and affected the relationship and trust of the GP, fostered by the GP refusing to act upon the informant's symptoms and information from a chiropractic examination. Other statements described a mix of presence and absence of the experience of being taken seriously. An informant told how she felt taken seriously when the GP referred her to a specialist clinic to give her the best treatment. She also told about the pronounced disappointment after waiting for months to see the specialist and then, at the consultation, getting the impression of not being expected but being arbitrarily treated with lack of general information and details of the diagnosis:

78-year-old former school teacher “I was not told how pronounced the osteoporosis was, he [the specialist] sat with those copies himself”

This experience was described as fostering a feeling of not being taken seriously because the informant had expected a thorough and informative consultation and clarity of the diagnosis.

*Acceptance of the diagnosis* was for some a matter of an immediate occurrence, for others it was found to be a struggle. An immediate occurrence of acceptance of the diagnosis was told as being a matter of trust toward the physician and health care system, or perceived fear of what could happen. The fear of falling, maybe have a fracture, and getting visible signs of osteoporosis, was described and interpreted to affect the acceptance of the diagnosis. The fear of falling led to precautionary actions such as using suitable shoes, not climbing on anything, or planning when shopping for groceries. One informant said:

72-year-old former housewife “I need my wheeled walker. I might take a wrong step. I have become even more afraid of falling after I've got osteoporosis. The thought of if I'll break the hip or femur frightens me”

The perceived fear of what could happen if a fracture occurred was described as fear of being dependent on someone else's assistance and no longer being able to lead an independent life. The image of the late Queen Ingrid was mentioned as well as the fear of coming to look like one's own mother or grandmother with visible signs of osteoporosis and daily pain. Osteoporosis was in some cases discovered at the Centre of Clinical and Basic Research, a private company located in the region. More than half of the informants had at some point been in contact with this company. At the time of inclusion for the current study all women were treated in the established health care system:

73-year-old former shop assistant “I'd rather be affiliated with a hospital. I'm probably a little authoritarian. Normally I don't have that much confidence in the system, but that is nevertheless where I feel safest”

Trust toward the health care system and the physician was found to affect acceptance of the diagnosis and life with osteoporosis. Descriptions of acceptance being a struggle appeared frequently with a considerable element related to the invisibility of osteoporosis and often related to the understanding of the result from the DXA scan as the visible “proof” of osteoporosis:

76-year-old former chemical engineer “I am really set on the idea that I will try to let it go as it goes. I'm not down in the red zone”

It was commonly found that the DXA scan result was solely viewed while omitting other risk factors and the fact that the scan is only one part of the assessment of increased risk of osteoporotic fractures. Acceptance could also be challenged by psychosocial factors in terms of osteoporosis being “a separate part” and not relating to one self or osteoporosis as something not spoken about. Stories of reluctance to “take the diagnosis in” as part of the current life circumstances were told in a context of refusing to think of osteoporosis because it was depressing and was perceived as a sign of getting old. It was important to stay independent and not become a burden on the family or the health care system. By keeping the diagnosis as “a separate part” not spoken about, the fear of being considered “whining” or sickly was avoided:

65-year-old secretary “I will not be whining as my mother and sister. I don't want to entertain my surroundings with me having osteoporosis”

Life in general, mental and physical well-being, was influenced by the way the diagnosis was handled by the individual.

*Being diagnosed* led to a need of understanding the diagnosis, being taken seriously, and accepting the diagnosis through development of an awareness of the impact of osteoporosis on everyday life. The subtheme, *being taken seriously*, appeared widely in the stories about being diagnosed. This was mainly as positive or negative experiences, but was also found as a combination of the two. It was widely spoken of in the context of past experiences, experiences during the current diagnosing process, and experiences following the diagnosis (related to the feeling of be taken care of and trust in the relationship with the physician) promoting either positive or negative attitudes toward the health care system and trust toward the physician. Thus, the subtheme, *acceptance of the diagnosis*, was closely connected to being taken seriously. This was interpreted to be a central aspect of being diagnosed. This was described to be formed by experiences in relation to trust toward the health care system and the physician, but also fear of falling and getting a bone fracture, understanding and weighting of the DXA scan result, and the importance of leading an independent life and not becoming a burden. Some of the informants accepted the diagnosis rapidly whereas others described it as a difficult struggle. Being diagnosed led to, on the one hand, anxiety regarding future expectations and, on the other hand, to feelings of being satisfied but slightly alarmed as long as it did go well.

### Being prescribed medical treatment

The interpretation of *being prescribed medical treatment* was supported in the text by descriptions of experiences related to two subthemes: *decision against the medical treatment* and *decision to pursue the medical treatment*. Shortly after diagnosis, informants in general focused on practical issues (such as how to take the medication and remembering to take the medication) which are a part of the process of achieving meaning and understanding of the diagnosis. Concerns of forgetting to take the medication, feeling of security, and faith in the medical treatment's ability to prevent deterioration and disability was described:

75-year-old former housewife “I take so many pills; just throwing it down. It serves no purpose to think about it. On Sunday night I put them on the nightstand. Thus, I have control. Otherwise I run the risk of forgetting it”

It was as easy as that for a minority of the informants.

*Decision to pursue medical treatment* was describing issues associated with making a choice that could be an immediate decision or a decision filled with worrisome thought. Thoughts, worries, and anxiety about the decision regarding the medical treatment were mainly described in relation to the comprehensive package leaflet in the medication package and possible side effects. One informant stood out by the richly described and recurring sense of responsibility and concerns about whether she made the right choice:

66-year-old former high school teacher “I have many thoughts, oh my God, is it harmful... do I make the right choice? I find it very difficult to choose”

These worrisome thought seemed to be enhanced by the experiences of not getting the information needed from the GP and other health care professionals. Moreover, information sought from other sources (others’ experiences or information through the Internet and likewise) did not always help.

Others described the total opposite experience when making the decision to take the medication:

68-year-old former medical Secretary “It is kind of automatic I take those pills like I take those for the blood pressure. Just like, I believe other people get the idea that they need a vitamin pill”

When the decision seemed to be straight on and not lead to deeper considerations, it was common that the informants were preoccupied with other issues (such as being widowed recently, other more urgent comorbidities, or an ill spouse to take care of).

*Decision against the medical treatment* informants described that potential side effects were worrisome, causing anxiety. Side effects of the medical treatment were described as own experiences, others’ experiences, and information in the comprehensive package leaflet in the medication package. For one informant it was a matter of private attitude toward medication in general when she decided against the medical treatment:

77-year-old former shop assistant “I am absolutely hysterical with medication, pills and such. Because, the pills helps treating one thing but it also harms something else. I cannot fill myself with all that crap”

*Being prescribed medical treatment* appeared to be a cognitive process of comprehension and meaning creation reflected in a decision-making process on whether to pursue or reject medical treatment. This process was associated with the individual's experiences, thoughts, and perception. Prominent in stories about handling practical issues were worries and need for information and knowledge about osteoporosis and anti-osteoporotic medications, side effects and discomfort, as well as attitudes and current life circumstances.

### Being on the path of learning to live with osteoporosis

Stories of understanding and meaning creation related to the new life circumstances with osteoporosis were described as experiences related to two subthemes: *the need to adapt* and *lifestyle changes*. To be on the path of learning to live with osteoporosis was interpreted to relate to approving and incorporating osteoporosis as a part of current life.

*The need to adapt* was found to be closely related to awareness and understanding of osteoporosis which initiated a process of decisions not to let the diagnosis control how life is lived, but instead sustain hope:

67-year-old former cleaning lady “I have always looked forward and I still do; it cannot do any good to put yourself away and sink into a chair because you have something”

Some informants adapted to living with osteoporosis shortly after the diagnosis. It came together with the acceptance of the diagnosis and decision to pursue the medical treatment. Some women described their experiences of living with osteoporosis to be completely non-intrusive to the point of “forgetting about the disease.” Others described osteoporosis as intrusive and affecting basic needs in daily life:

79-year-old former nurse “of course I do not have much desire for food. I eat much less than usual, but I'm not doing as much exercise”

The descriptions of osteoporosis as being intrusive were in some cases later on described in the light of a new context of meaning creation. Thereby an awareness of the need to actively adapt to the new circumstances occurred.

*Lifestyle changes* was described as stories of being on the path of learning to live with osteoporosis, as becoming aware of the need to focus on current life circumstances, and considerations to be taken as an ongoing need for planning daily activities. Active decisions about small changes of habits were described:

66-year-old former librarian “Even the days when it feels like I don't want to go for a walk – I'll do it anyway. I love to sit in the shade, but I am starting to sit more in the sun”

Focus was turned, information and good advice were considered, and new recipes and physical activities were tested. Focus on enjoying life despite having osteoporosis led to bright ideas of how to be physically active and sometimes to take on new challenges:

67-year-old former nursing assistant “I'm really proud of myself of being a new member of the rowing club”

In contrast, only one informant's story did not include descriptions about lifestyle changes. She was preoccupied with other concurrent health problems. Her story contained descriptions of acceptance of the diagnosis and how she pursued the medication. That was how she decided to live with osteoporosis.

*Being on the path of learning to live with osteoporosis* was interpreted as a cognitive process incorporating and formed by processes of being diagnosed and being prescribed medical treatment when adapting to the new life circumstances. This was a continued cognitive process of understanding and meaning creation that made an awareness of *the need to adapt*, as it stands in the subtheme. *Lifestyle changes* were based on new knowledge that was obtained through various sources of knowledge of osteoporosis. This led to a change and a decision to focus on how to live with osteoporosis by testing advice, bright ideas, and adapting to a new lifestyle with confidence and pride.

By telling about experiences of everyday life the informants constructed stories of their lives when they were living with osteoporosis requiring treatment, but before anyone known osteoporotic fracture had occurred.

## Discussion

This study illuminated women's experiences of living with a new osteoporosis diagnosis during the first 6 months after diagnosis when treatment was prescribed. The study showed living with a new diagnosis was a cognitive process of understanding and meaning creation when developing an awareness of the impact of osteoporosis on everyday life and the need to adapt to the new life circumstances. The findings will be discussed in relation to Antonovsky's theory of Sense of Coherence (SOC) (Antonovsky, 1979) and other literature, as well as research findings. When the individual is exposed to stress through life events such as being diagnosed, a cognitive process of comprehension and meaning creation is initiated, and according to Antonovsky, this affects the individual's SOC. SOC is a salutogenic orientation concerning how humans interpret and relate to life circumstances (Antonovsky, 1979).

The findings of this study were, to some extent, in line with another qualitative study of everyday life of patients with osteoporosis (Nielsen et al., 2013). Nielsen (2013) found that those not suffering from severe osteoporosis or fracture experienced emotional difficulties when handling osteoporosis. In comparison, it was found in the current study, that those who described earlier experience of critical illnesses, previous experiences of not being taken seriously during contact with the health care system, and not having osteoporotic symptoms at the current state, described these experiences in a context of a need for more information. Moreover, they were relating the need for more information to feelings of being taken care of and experiencing trust in the relationship with the physician; it appeared that these women had difficulties in handling the diagnosis. In relation to Antonovsky's theory (Antonovsky, 1979), stressors in the context of lack of information may be seen as disrupting the ongoing development of SOC in relation to comprehending, managing, and finding meaning in the new life circumstance. These types of stressors were interpreted to be core issues in the current study. The women told about experiences of worries or anxiety when they sought to understand the diagnosis, the medical treatment, and how to live with osteoporosis. This finding is somewhat in line with the study conducted by Weston et al. (Weston et al., 2011), who found the anxiety level to be influenced by the women's interpretation of core meanings of the diagnosis and the sense of it in relation to their current lives. Furthermore, it is argued that the SOC measuring instrument may be a useful tool to gain a deeper understanding of patient circumstances, enabling it to be used in the planning of the individual nursing care (Langius, Bjorvell, & Antonovsky, 1992). The SOC measuring instrument reflects the individual's coping capacity and subjective well-being and as such is useful in identifying those who are at risk of poorer quality of life and could be in need of supportive care (Mendel, Bergenius, & Langius, 2001). In the present study, it was found that fear of future perspectives of falling and having an osteoporotic fracture affected acceptance of the diagnosis; moreover fear was found to affect the decision regarding the medical treatment. The propensity to fall is generally known to be increased with increasing age and it is related to chronic diseases and the individual's nutritional status as well; these factors are often associated with hip-fracture. Appetite for food may be an important factor to be aware of when preventing falls and fracture, since appetite for food has been found to mirror older adults health condition, nutritional status, and the general life-situation (Mahler & Sarvimaki, 2012).

According to Antonovsky, development of SOC requires an opening up of a process of developing understanding and meaning creation when making decisions related to new life circumstances (Antonovsky, 1979). In the present study, it was found that when medical treatment was prescribed, the cognitive process of comprehension and meaning creation were reflected in the descriptions of decision-making regarding whether to pursue or reject medical treatment. In this process, an individual's perception may be a mediating factor and therefore important when dealing with one of the most urgent problems associated with osteoporosis; namely the challenges of non-compliance and persistence. In the current study all women did fill their prescriptions but some chose to stop treatment. Additionally, in a large national register-based study, we found that 38.7% stop treatment early (Hansen, Pedersen, Konradsen, & Abrahamsen, 2013). These results are rather low in an international comparison (Kanis et al., 2013; Landfeldt, Strom, Robbins, & Borgstrom, 2012). But still, many challenges relating to this issue remain in Denmark as elsewhere. In the current study, it seemed that discontinuation of the medical treatment was influenced by the interpretation of lack of information, experiences of not *being taken seriously* and experiences of side effects or discomfort. This is in line with a focus group study examining viewpoints of patients and providers (Iversen, Vora, Servi, & Solomon, 2011) where patients identified a lack of knowledge about osteoporosis, dissatisfaction with their physician visits, side effects, and difficulties with the medication instructions, as factors affecting the medical adherence.

Despite the discussed difficulties related to experiences of living with a newly diagnosed osteoporosis, the majority of the women were on the path to learning to live with osteoporosis with minor lifestyle changes. This may be seen to reflect the health risk awareness in line with the findings in the Swedish study (Hjalmarson et al., 2007). Also, in the current study it may be a combination of certain meaning-creating events that lead to life style changes in a context of finding new enjoyable forms of physical activity, together with adaptation to current life circumstances.

The study comprised 15 informants. This number is seen as strength of the study, because an excessive number of informants in a qualitative study may lead to superficial results without an in-depth investigation (Kvale & Brinkmann, 2009; Malterud, 2001). It could be assumed that women of younger age would have expanded and elaborated the themes. Furthermore, the women participated voluntarily, and it is always a possibility that those who did not wish to participate differ from those who chose to participate.

Individual interviews were chosen in order to explore the individual perspective in depth. The role of the interviewer (first author) may have affected the interviews, because the informants knew that she was a nurse. In addition, several of the women spontaneously described that they had chosen to participate in the project to have the opportunity to gain a better understanding of osteoporosis. One woman described that she did not talk to anybody else about osteoporosis, so it was helpful for her to be able to talk about her thoughts and concerns with someone who was trained in this area.

## Conclusion and implications

The phenomenological hermeneutic approach provided rich data describing experiences and relevant issues for women diagnosed with osteoporosis, which enrich the current knowledge in this field. An important finding in this study is that women handle osteoporosis in different ways. This is very much influenced by positive or negative experiences of the diagnosis process and seems to affect the acceptance of the diagnosis and living with osteoporosis in general. The findings suggest a need for improved support for the women to gain understanding of their diagnosis and the risk of osteoporotic fracture as well as to learning to live with osteoporosis. More research could also be done from the health promotion perspective, for example, further exploring SOC related to acceptance, interpretation of the diagnosis and the medical treatment, and learning to live with osteoporosis.
